# Medical Items

**Published:** 1913-10

**Authors:** 


### MEDICAL ITEMS

### A FAMILIAR FORM OF CYSTITIS.

There is a form of cystitis quite familiar to the general practitioner. It occurs in females, old and young, with apparently normal pelvic organs, generally after a chilling. There is an abrupt onset with frequent micturition, tenesmus, and perhaps dysuria. The acid urine contains the infecting organism, usually a colon bacillus, pus, and often blood. Rest in bed, local warmth, light diet, free catharsis and sanmetto are the measures employed, and in a few days the severity of the attack subsides, and generally in two or three weeks the patients are as well as ever.

